# The PI3K-Akt-mTOR pathway regulates Aβ oligomer induced neuronal cell cycle events

**DOI:** 10.1186/1750-1326-4-14

**Published:** 2009-03-16

**Authors:** Kiran Bhaskar, Megan Miller, Alexandra Chludzinski, Karl Herrup, Michael Zagorski, Bruce T Lamb

**Affiliations:** 1 Department of Neurosciences, Cleveland Clinic Foundation, Cleveland, OH, USA; 2 Department of Chemistry, Case Western Reserve University, Cleveland, OH, USA; 3 Cleveland Clinic Lerner College of Medicine, Cleveland, OH, USA; 4 Department of Cell Biology and Neuroscience, Rutgers University, Piscataway NJ, USA; 5 Department of Genetics, Case Western Reserve University, Cleveland, OH, USA; 6 Department of Neurosciences, Case Western Reserve University, Cleveland, OH, USA

## Abstract

Accumulating evidence suggests that neurons prone to degeneration in Alzheimer's Disease (AD) exhibit evidence of re-entry into an aberrant mitotic cell cycle. Our laboratory recently demonstrated that, in a genomic *amyloid precursor protein *(*APP*) mouse model of AD (R1.40), neuronal cell cycle events (CCEs) occur in the absence of beta-amyloid (Aβ) deposition and are still dependent upon the amyloidogenic processing of the amyloid precursor protein (APP). These data suggested that soluble Aβ species might play a direct role in the induction of neuronal CCEs. Here, we show that exposure of non-transgenic primary cortical neurons to Aβ oligomers, but not monomers or fibrils, results in the retraction of neuronal processes, and induction of CCEs in a concentration dependent manner. Retraction of neuronal processes correlated with the induction of CCEs and the Aβ monomer or Aβ fibrils showed only minimal effects. In addition, we provide evidence that induction of neuronal CCEs are autonomous to primary neurons cultured from the R1.40 mice. Finally, our results also demonstrate that Aβ oligomer treated neurons exhibit elevated levels of activated Akt and mTOR (mammalian Target Of Rapamycin) and that PI3K, Akt or mTOR inhibitors blocked Aβ oligomer-induced neuronal CCEs. Taken together, these results demonstrate that Aβ oligomer-based induction of neuronal CCEs involve the PI3K-Akt-mTOR pathway.

## Background

Alzheimer's disease (AD) is the most common form of senile dementia and is a leading cause of disability and death [1,2]. Currently, there are no reliable biomarkers or therapeutic agents to detect or prevent AD, respectively. A definitive diagnosis of AD requires the demonstration of distinctive brain pathology, including extracellular deposits of the beta-amyloid (Aβ) peptide in senile plaques and intracellular aggregates of the microtubule-associated protein, tau, in neurofibrillary tangles. Another invariant feature of the disease includes substantial neuronal cell loss in discrete brain regions, although the mechanisms underlying the neurodegeneration remain unclear.

Increasing evidences suggest that aberrant neuronal cell cycle re-entry may precede the regional neurodegeneration observed in AD. First, several reports have demonstrated the expression of cell cycle proteins and DNA synthesis in nerve cells susceptible to death in AD [3-6]. Second, brain tissue from individuals with mild cognitive impairment (MCI), believed to be the clinical predecessor to AD [7], reveals the evidence of cell cycle events (CCEs) even in the absence of substantial AD pathology [3]. Third, neuronal CCEs are observed in a variety of transgenic mouse models of AD [3,8]. In particular, we have observed that the genomic-based *amyloid precursor protein *(*APP*) transgenic mouse model of AD, R1.40, exhibits significant re-expression of cell cycle proteins and DNA replication [9]. The neuronal populations involved and the timing of the events accurately recapitulate the selective neuronal vulnerability observed in human AD [3,10]. Finally, we recently reported that neuronal CCEs in the R1.40 mouse model occur in the absence of Aβ deposition and yet are dependent upon the amyloidogenic processing of the APP by beta-secretase [9,11]. Together, these data suggest that APP and its cleavage at the beta-secretase site play a direct role in the induction of neuronal CCEs. The exact nature of the neurotoxic substance is still unknown.

Recently, a number of findings have implicated soluble, macro- or micromolecular assemblies of Aβ, termed Aβ oligomers, in the pathogenesis of AD. Small, secreted Aβ oligomers (dimers and trimers), are capable of inhibiting long-term potentiation (LTP) in hippocampal slice cultures, an effect blocked by immunoneutralization or inhibition of oligomerization [12-14] and exposure of primary neurons to synthetic preparations of Aβ oligomers (also referred to as Aβ-derived diffusible ligands, ADDLs) results in significant synaptotoxicity including decreased dendritic spine density [15,16] (reviewed in [17,18]). It has also been reported that the appearance of a 56 kDa Aβ species (Aβ*56) observed in the Tg2576 mouse model of AD correlates with the timing of memory impairments in this model. Further, Aβ*56 disrupts memory when purified and injected into the brains of rats [19]. Finally, our recent studies have demonstrated that crude Aβ oligomers, but not Aβ monomers, can directly induce neuronal CCEs in primary cortical neurons [9]. Although the Aβ oligomers promote diverse biological consequences, the relevant oligomeric species, the biological mechanisms underlying these effects and the relationship among the various phenotypic consequences remains to be determined.

Increasing evidence suggests that the phosphatidylinositol-3-OH kinase (PI3K)-Akt-mTOR signaling pathway is directly impacted by Aβ exposure and is altered in AD brains. First, Aβ oligomers have been shown to modulate expression and density of insulin receptors that mediate growth and cell survival through the modulation of PI3K-Akt pathway [20] (reviewed in [21]). Second, significant increases in the levels of phosphorylated Akt substrates such as mTOR (Ser2448) and decreased levels of cell-cycle inhibitors (p27^kip1^) are found in AD temporal cortex when compared to controls [22]. Third, studies in transgenic mouse models and patients with AD have documented alterations in mTOR/p70S6K signaling by Aβ exposure [23]. Fourth, TOR activation has been demonstrated to enhance tau-induced neurodegeneration in a cell-cycle dependent manner in a tauopathy model of *Drosophila *and that ectopic TOR activation drives cell cycle and apoptosis in post-mitotic neurons [24]. Finally, the levels of mTOR and its downstream targets eukaryotic translation initiation factor 4E binding protein 1 (4E-BP1), as well as the eukaryotic translation elongation factor 2 (eEF2) and eEF2 kinases are up regulated in the AD brain [25]. Together, these studies suggest that the components of insulin receptor and PI3K-Akt-mTOR pathway are affected in AD and correlate with altered cell cycle related events.

Based on the above studies, we hypothesized that Aβ oligomers alter the PI3K/Akt/mTOR pathway within neurons, and in turn promotes the expression of cell cycle proteins and the induction of neuronal CCEs. To test this hypothesis, we exposed primary cortical neurons to size-exclusion column (SEC) purified monomeric and oligomeric samples of the Aβ_1–42 _peptide. Our results demonstrate that Aβ oligomers induce neuronal process retraction and neuronal CCEs in a concentration dependent manner, while the monomeric samples showed no response. Furthermore, while the presence of CCEs correlated with neuronal process retraction in the cultures, inhibition of either the PI3K, Akt or mTOR pathway substantially blocked the neuronal CCEs, while only inhibition of the PI3K pathway significantly blocked neuronal process retraction. These studies help identify new components of the biological pathways responsible for the diverse pathological consequences of Aβ oligomers.

## Results

### Preparation of Aβ monomers and oligomers

**Figure 1:**
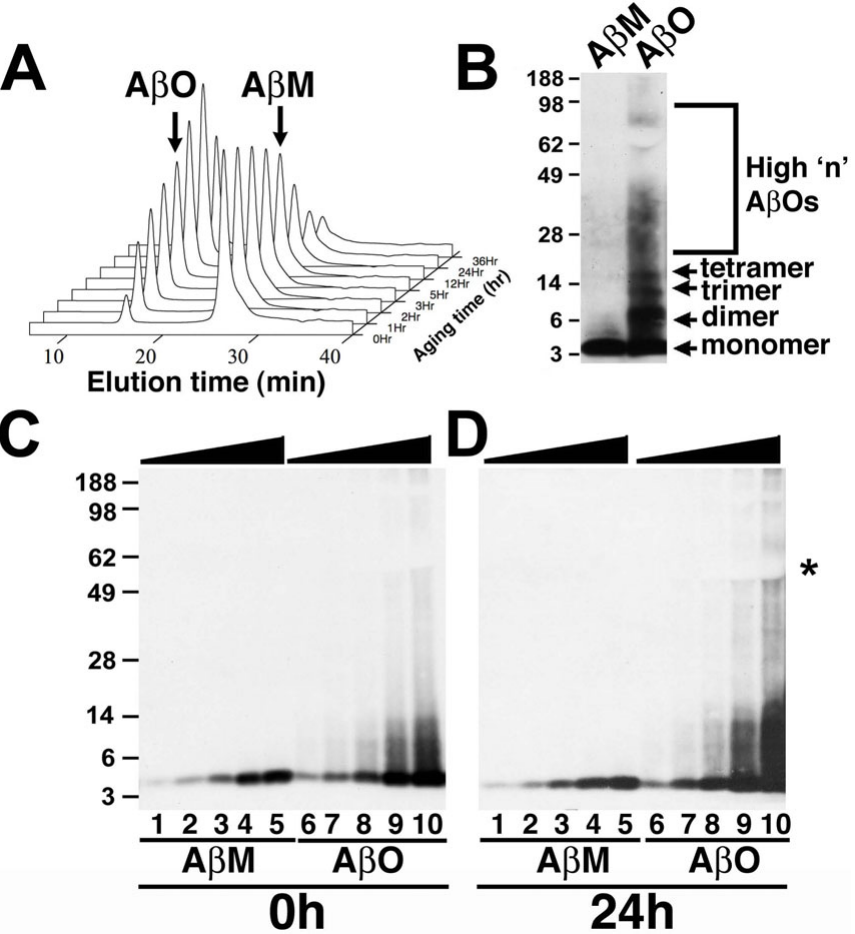
**Preparation and characterization of Aβ monomers and Aβ oligomers**. **A**. Preparation of Aβ monomers and Aβ oligomers. Synthetic Aβ peptides were aged for various amounts of time in phosphate buffer followed by SEC fractionation, yielding two major peaks corresponding to Aβ oligomers (AβO) and Aβ monomers (AβM), with Aβ oligomers eluting at 12–15 min and Aβ monomers eluting at 25–30 min. With increased aging, the relative amount of Aβ oligomers increased to a maximum at 24 h. The Z-axis is the relative absorption intensity at 220 nm. **B**. Western blot analysis of SEC fractionated *in vitro *preparations of Aβ monomers (AβM) and Aβ oligomers (AβO) with the Aβ specific antibody 6E10, revealed a single, 4.5 kDa monomeric band for AβM and monomer, dimers, trimers, tetramers and large molecular weight oligomers that run between 28–90 kDa (high 'n' AβOs) for AβO. **C, D**. Western blot analysis of tissue culture media with 0.2 μg/ml (lanes 1 and 6), 0.4 μg/ml (lanes 2 and 7), 0.8 μg/ml (lanes 3 and 8), 2.0 μg/ml (lanes 4 and 9) and 4.0 μg/ml (lanes 5 and 10) of AβM and AβO at the beginning (time = 0 h, **C**) or end (time = 24 h, **D**) of the experiment revealed a similar migration pattern of AβM and AβO at both time points. Asterisk indicates the location of abundant proteins in the B27 serum-free supplement.

To examine the role of specific Aβ species in induction of neuronal CCEs, we utilized purified synthetic preparations of Aβ. Aβ oligomers were prepared by established protocols via disaggregating Aβ peptides in sodium hydroxide, "aging" of Aβ for various time points in PBS and finally purification of Aβ monomers and Aβ oligomers via SEC fractionation (Figure 1A and 1B). Aβ prepared in this manner yielded consistent, homogeneous monomer and oligomer peaks with an increased proportion of oligomers upon longer incubation times (Figure 1A). As the 3-hour incubation time yielded relatively equal concentrations (peak intensities) of monomeric and oligomeric Aβ species without forming protofibrils/fibrils, SEC fractions from these conditions were utilized for all subsequent experiments.

The presence of monomeric and oligomeric Aβ species in the SEC fractions was confirmed by Western blot analysis. Consistent with previous findings [26-28], detection with monoclonal Aβ antibody 6E10 revealed that the monomeric preparations contained 4.5 kDa reactive Aβ monomers, while the oligomeric preparations contained both the larger molecular weight and monomeric Aβ (Figure 1B). Quantification of the relative proportion of the different sized Aβ species in the SEC fractions demonstrated that the Aβ monomeric fractions contained 100% of Aβ monomers, while the Aβ oligomeric fractions contained 26% of Aβ monomers, 24% of Aβ dimers, 20% of Aβ trimers, 18% of Aβ tetramers and 12% of higher molecular weight (> 20 kDa) Aβ oligomers.

### Aβ oligomers induce neuronal CCEs

**Figure 2:**
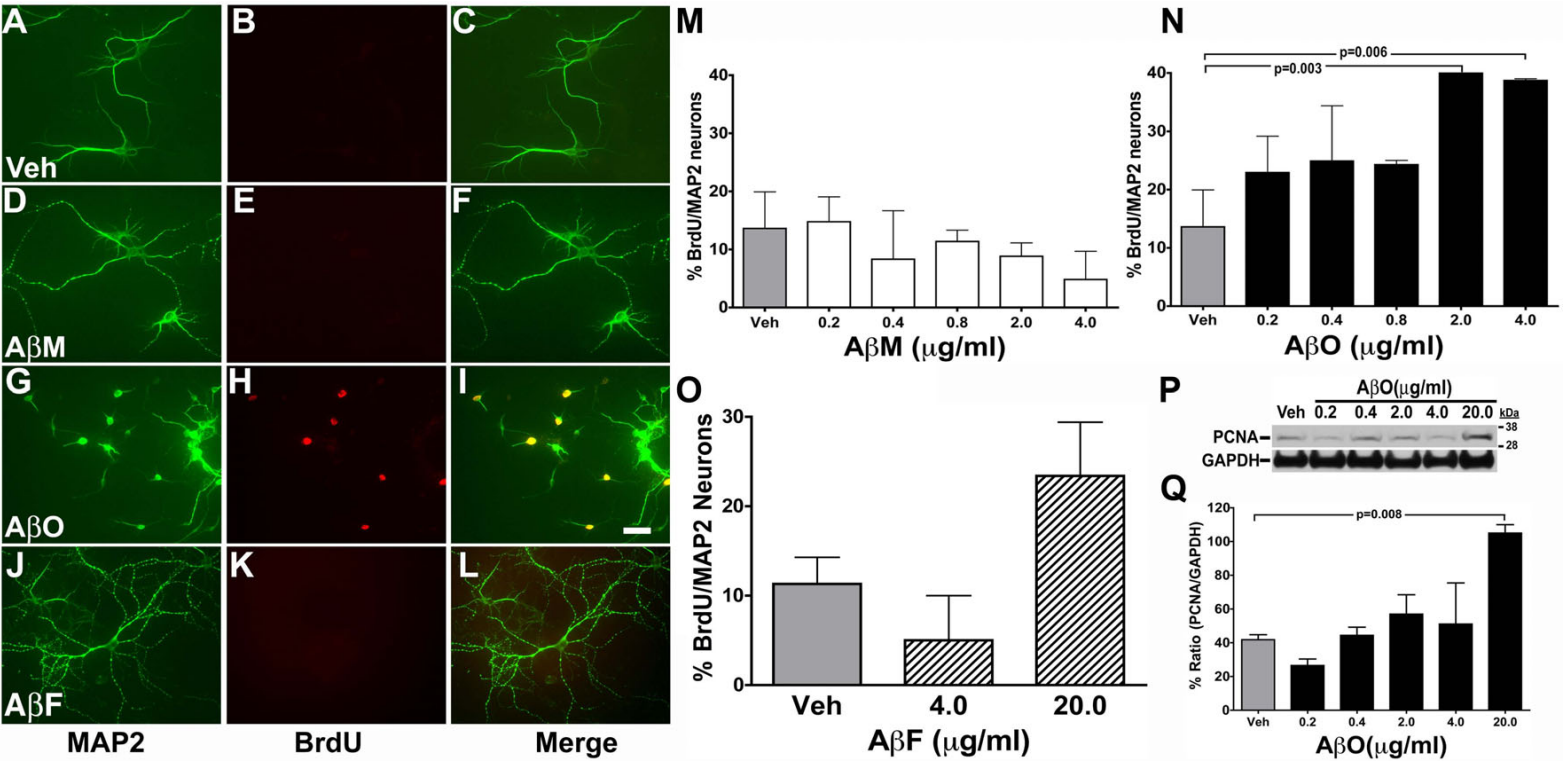
**Purified Aβ oligomers induce BrdU incorporation in primary neurons**. Cultured primary cortical neurons (21 DIV) were treated with vehicle (Veh) **A-C**, 0.4 μg/ml of Aβ monomers (AβM) **D-F **or 0.4 μg/ml of Aβ oligomers (AβO) **G-I **or 0.4 μg/ml Aβ fibrils (AβF) **J-L **in presence of BrdU for 24 h. Cells were fixed and immunostained with antibodies against MAP2 (**A**, **D**, **G and J**) and BrdU (**B**, **E**, **H and K**) demonstrating the induction of BrdU incorporation in MAP2 positive cells (neurons) with Aβ preparations, but not with AβM, AβF or vehicle control (merged images in **C**, **F**, **I and L**). Scale bar, 10 μm. **M-O **Quantification of the percentage of BrdU positive/MAP2 positive cells in vehicle, AβM, AβO and AβF treatment groups revealed a statistically significant (p = 0.003 for 2.0 μg/ml; p = 0.006 for 4.0 μg/ml; unpaired *t *test; mean ± SEM; n = 3 independent experiments) increase in the percentage of BrdU/MAP2 positive cells upon exposure to AβO, but not AβM or AβF, compared to vehicle control. **P**. Western blot analysis of detergent soluble cell lysates from neurons exposed to different concentrations of Aβ with an antibody against the S-phase cell cycle protein, PCNA, and an antibody against the protein loading control, GAPDH. **Q**. Quantification of the Westernblot for PCNA (relative to GAPDH) revealing a statistically significant (p = 0.008 for 20 μg/ml) increase in the PCNA expression in the 20 μg/ml AβO treatment group. PCNA/GAPDH ratio at 20 μg/ml AβO treatment was assigned as 100%.

To examine whether SEC purified Aβ monomers and Aβ oligomers were capable of inducing neuronal CCEs, we exposed 21 DIV primary cortical neurons to different concentrations (0.2 to 4.0 μg/ml) of the SEC fractions for 24 hours in the presence of BrdU. We selected 21 DIV for all the treatments because majority of neurons at that point are mature and terminally differentiated. However, we have consistently observed that ~5–10% of the MAP2 positive neurons display basal level of BrdU incorporation in 21 DIV cultures (Figure 2M–O Veh). These results are consistent with previous findings on mouse [29,30] and rat cortical neurons at 5–10 DIV [31]. At present, whether this baseline induction of BrdU incorporation is due to continued division of a small number of neuronal stem cells or accounted for by the de-differentiation of a low number of non-neuronal cells remains unclear.

Given concerns that the conformation of the Aβ species could alter during this treatment paradigm, we first examined the tissue culture media for the sizes of the Aβ species via Western blot analysis prior to and following exposure to both Aβ monomers and oligomers. Detection of Aβ using 6E10 revealed that both monomeric and oligomeric Aβ species exhibited little alterations in mobility at the 24 hour time point (Figure 1C-24 h) when compared to the starting material (Figure 1C-0 h) at all of the concentration ranges examined. These results demonstrate that the Aβ monomers and oligomers are relatively stable during the exposure period.

The effect of exposure to Aβ monomer and oligomers on neuronal CCEs was determined via dual immunofluorescence for the neuronal maker MAP2 and BrdU. These studies revealed an increase in nuclear BrdU incorporation in Aβ oligomer treated neurons (Figure 2G–I), but not in Aβ monomer treated neurons (Figure 2D–F), when compared to vehicle treated neurons (Figure 2A–C). Quantification of the results indicated a 2- to 5-fold increase in BrdU incorporation in the Aβ oligomer treatment group (Figure 2N) when compared to the basal level of BrdU incorporation (13 ± 6%) in vehicle treated groups (Figure 2M–O – Veh). There was a concentration dependent increase in BrdU incorporation upon exposure of neurons to Aβ oligomers that reached statistical significance at 2.0 to 4.0 μg/ml of oligomers (Figure 2N). By contrast, different concentrations of SEC purified Aβ monomers did not alter the total number of BrdU positive neurons when compared to vehicle treated groups at any of the concentrations examined (Figure 2D–F and 2M). Interestingly, the concentration ranges of Aβ oligomers exhibiting effects on BrdU incorporation in the current studies are similar to the previously reported effects on synaptotoxicity [15].

To examine whether the effects of oligomeric Aβ on neuronal CCEs could be due to contamination of the purified oligomeric preparations with extremely low levels of Aβ fibrils, that were below our limits of biochemical detection, Aβ fibrils were also prepared [32] and exposed to 21 DIV primary cortical neurons. Exposure of neurons to Aβ fibrils at a concentration (4.0 μg/ml) at which Aβ oligomers exhibit robust induction of neuronal CCEs, demonstrates that unlike Aβ oligomers, Aβ fibrils did not induce any statistically significant alterations in BrdU incorporation (Figure 2J–L and 2O). Furthermore, we also examined the effects of exposure to even higher concentration of Aβ fibrils (20 μg/ml), based on the earlier reports that Aβ fibrils can induce cell cycle events at higher concentrations [30]. Exposure of neurons to the higher concentration of Aβ fibrils resulted in a trend towards increased BrdU incorporation that did not reach statistical significance (Figure 2O). Our results are in agreement with earlier reports that ~4.0 μg/ml of Aβ_25–35 _fibrils did not induce increased numbers of BrdU positive neurons [29], while ~40 μg/ml of Aβ_1–42 _fibrils induced a two-fold increase in the percentage of neurons incorporating BrdU [30]. Taken together, these results demonstrate that Aβ oligomers are a more potent inducer of neuronal cell cycle events than either Aβ monomers or Aβ fibrils.

To confirm that Aβ oligomer treatment induced aberrant neuronal CCEs, cell lysates were prepared at different exposures and examined for induction of the Proliferative Cellular Nuclear Antigen (PCNA), a marker of the S-phase of cell cycle. Consistent with the results from the quantification of BrdU incorporation, treatment with increasing concentrations of Aβ oligomers induced a concentration-dependent increase in the levels of PCNA in the soluble cell lysates from cortical neurons (Figure 2P). Western blot qauntification of PCNA expression after exposure of neurons to different concentrations of Aβ oligomers and normalization to the levels of GAPDH, revealed a concentration dependent increase in the levels of PCNA (Figure 2Q), that reached statistical significance for the highest concentration of Aβ exposure (41.82 ± 2.9 for vehicle versus 105 ± 5.0 for 20 μg/ml Aβ oligomer, p = 0.008). By contrast, exposure to Aβ monomers did not exhibit the same increase in the levels of PCNA (data not shown). Taken together, these results demonstrate that purified preparations of stable Aβ oligomers induce CCEs in primary cortical neurons in a concentration dependent manner that is not observed with preparations of Aβ monomers or Aβ fibrils.

### Aβ oligomers induce coincident neuronal CCEs and dendritic loss

**Figure 3:**
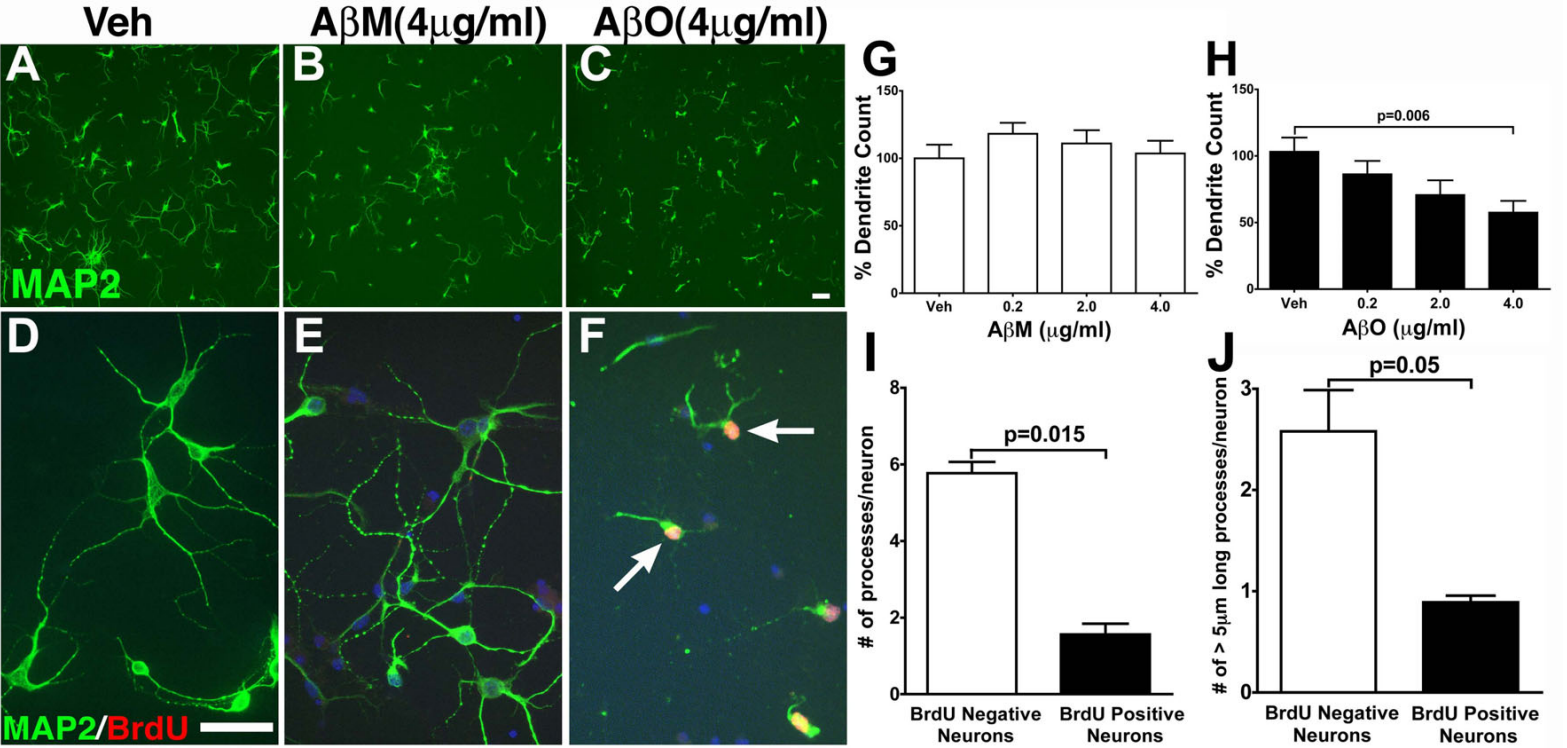
**Aβ oligomers induce loss of MAP2 positive processes in primary neurons coincident with BrdU incorporation**. Cultured primary cortical neurons (21 DIV) were treated with vehicle (Veh, **A **and **D**), 4 μg/ml Aβ monomers (AβM, **B **and **E**) or 4 μg/ml Aβ oligomers (AβO, **C **and **F**) for 24 hours. Following fixation, cells were immunostained with antibodies against MAP2, revealing several long MAP2 positives processes per cell in the vehicle treatment group, a modest reduction in the number of MAP2 positive processes in the AβM treatment group and a dramatic reduction in the number and length of the MAP2 processes in the AβO treatment group. Scale bar, 10 μm. MAP2 positive neurons with shorter processes displaying BrdU incorporation (arrows in F). **G-H**. Quantification of MAP2 positive dendrites following exposure to Veh or different concentrations of AβM (**G**) and AβO (**H**) via automated image processing revealed a statistically significant decrease in MAP2 positive processes (p = 0.006; Veh versus AβO-4 μg/ml; unpaired *t *test; mean ± SEM; n = 3 independent experiments) when compared to either vehicle or AβM. **I-J**. Quantification of the total number (**I**) of MAP2 positive processes as well as the number of processes longer than 5 μm per cell (**J**) in both BrdU positive and BrdU negative neurons revealed a statistically significant reduction in both number of MAP2 + processes (p = 0.015; mean ± SEM; unpaired *t *test; n = 3) as well as the number of processes longer than 5 μm (p = 0.05; mean ± SEM; unpaired *t *test; n = 3) in BrdU positive cells when compared to BrdU negative cells.

In analyzing the effect of Aβ oligomers on cortical neurons, a decrease in the number of MAP2 staining neuronal processes was consistently observed (see Figure 2G–I and Figure 3A–F). In general, control neurons exhibited 4–5 MAP2+ processes per cell, while those exposed to Aβ oligomers exhibited an apparent concentration dependent decrease in the number of MAP2+ processes together with increased nuclear BrdU staining. Quantification of these results was performed by examining the total number of dendrites per field in 5 random fields per treatment using a macro ("Dendrites") in the ImagePro Plus™ image analysis software (Materials and Methods). The data revealed that the number of dendrites was significantly reduced upon exposure to increasing concentration of Aβ oligomers (Figure 3H) when compared to either vehicle or increasing concentrations of Aβ monomer treatment (Figure 3G) or Aβ fibrils (data not shown). These results are consistent with earlier reports demonstrating that Aβ oligomer exposure induced a loss of the spine marker, drebrin, as well as dramatic changes in spine morphology and density in mature hippocampal cultures [15]. Similar findings were obtained upon Aβ exposure of 7 DIV cortical neuronal cultures (data not shown). Interestingly, none of the Aβ treatments, even at the highest concentrations, altered the total number of MAP2 positive cells nor exhibited a significant alteration in neuronal cell number as determined by measuring the formation of formazan from reduced MTT (3-(4,5-Dimethylthiazol-2-yl)-2,5-diphenyltetrazolium bromide) in an *in vitro *cell-death assay (data not shown). These results suggests that consistent with previous data, Aβ oligomer exposure at sub-nanomolar concentrations induces synaptic and dendrite loss in the absence of robust neurodegeneration [15,33].

To begin to examine whether the loss of MAP2+ processes and induction of neuronal CCEs were part of a coordinated response to exposure to Aβ oligomers, we quantified the number and length of MAP2+ processes in both BrdU positive and BrdU negative cells exposed to 2.0 μg/ml Aβ oligomers. Interestingly, there was a 70% reduction in the total number of MAP2+ processes in BrdU positive neurons when compared to BrdU negative neurons (Figure 3F and 3I). In addition, the number of neurons with MAP2+ processes 5 μm or longer was reduced by 60% in BrdU positive neurons when compared to BrdU negative neurons (Figure 3F and 3J). Together, these results demonstrates that the loss of MAP2+ processes is correlated with the induction of neuronal CCEs and suggests that both phenotypes are likely part of a coordinated biological response of cortical neurons to Aβ oligomer exposure.

### Cell-autonomous induction of CCEs and process retraction in R1.40 (R/R) neurons

Our results demonstrate that neuronal CCEs and process retraction can be induced by exposure of wild-type neurons to increasing concentrations of exogenous Aβ oligomers. To examine whether these events are cell autonomous to neurons in the R1.40 mouse model of AD, primary embryonic cortical neurons derived from R1.40 mice (R/R) and wild-type C57BL/6 controls (WT) were prepared and examined for alterations in basal levels of neuronal CCEs and the process length.

**Figure 4:**
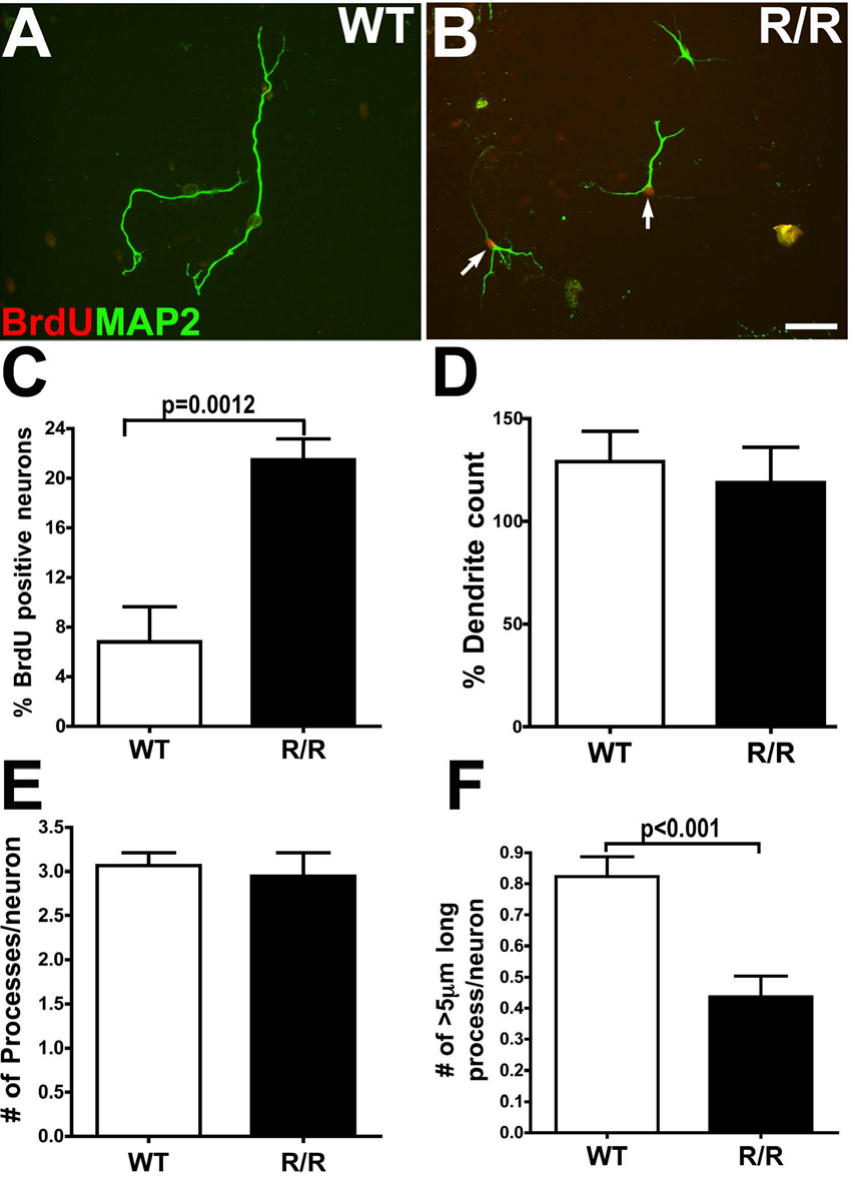
**Cell autonomous induction of BrdU and MAP2 process loss in primary neurons from the R1.40 transgenic mouse model of AD**. Cultured primary cortical neurons (21 DIV) from non-transgenic (WT, **A**) and homozygous R1.40 (R/R, **B**) mouse embryos were incubated with fresh NB media in presence of BrdU for 24 h. Following fixation, cells were immunostained with antibodies against MAP2 (green in **A **and **B**) and BrdU (red in **A **and **B**) revealing elevated incorporation of BrdU and a decrease in the length of MAP2 positive processes in the R/R cultures. **C**. Quantification of the percentage of BrdU positive/MAP2 positive cells demonstrated a statistically significant (p = 0.0012; unpaired *t *test; mean ± SEM; n = 4 independent cultures) increase in R/R cultures when compared to WT cultures. **D-F**. Quantification of the total dendrite count (total area occupied by all MAP2 positive dendrites per a given field/image) (**D**) and number of MAP positive processes per cell (**E**) were not significantly different between WT and R/R cultures, while the number of MAP2 positive processes longer than 5 μm per cell (**F**) were significantly (p < 0.001; unpaired *t *test; n = 4 independent cultures) reduced in the R/R cultures when compared to the WT cultures.

To examine the baseline levels of neuronal CCEs in the R/R neurons, BrdU immunofluorescence was performed as described above. The R/R neurons readily incorporated BrdU compared to neurons derived from WT controls (Figure 4A–B). Notably, there was a statistically significant increase in the number of BrdU positive cells in the R/R cultures when compared to controls (21.48 ± 1.67 versus 6.8 ± 2.8) (Figure 4C). Similar to our findings of wild-type neurons exposed to exogenous Aβ oligomers, a majority of R/R neurons with short MAP2+ processes also incorporated BrdU. The total number of process in BrdU+ vs BrdU- neurons was not significantly different, but the length of the processes in BrdU+ neurons is significantly shorter than those from BrdU – neurons (data not shown). A total of approximately 20–25% of the R/R neurons displayed BrdU incorporation, which is statistically significant and comparable to the levels observed in WT neurons exposed to 200 nM of exogenous Aβ oligomers (Figure 2K).

We then quantified the total dendrite count, total number of processes per cell and total number of processes that are 5 μm or longer from WT and R/R primary neurons. The data revealed no statistically significant differences in total dendrite count (Figure 4D) and total number of processes per neuron (Figure 4E) when compare to WT controls. However, the number of processes 5 μm or longer were significantly reduced in R/R neurons compared to control (0.43 ± 0.06 versus 0.83 ± 0.06) (Figure 4F), suggesting that majority of R/R neurons have an equal number but shorter MAP2+ processes when compared to controls. Together, these results suggest that primary cortical neurons derived from R/R mice exhibit cell autonomous induction of baseline neuronal CCEs and process retraction.

### Production of Aβ oligomers by R1.40 (R/R) neurons

**Figure 5:**
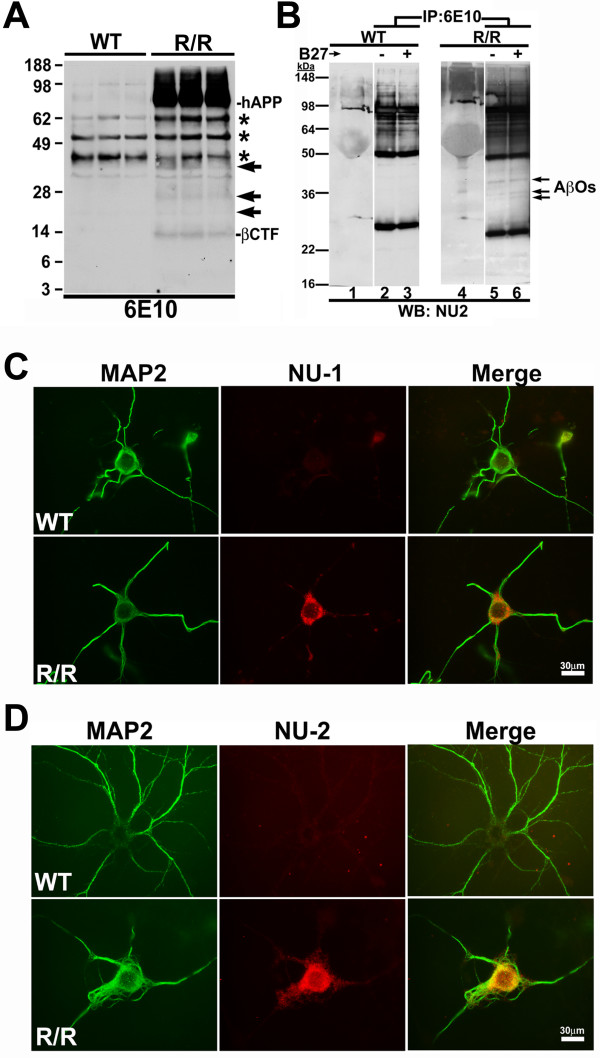
**Presence of cell-associated and secreted oligomeric Aβ assemblies in primary neurons from the R1.40 transgenic mouse model of AD**. **A**. Western blot analysis of detergent soluble cell extracts of from cultured primary cortical neurons (21 DIV) from non-transgenic (WT) and homozygous R1.40 (R/R) mice revealed the presence of numerous bands migrating between 14 and 38 kDa (arrows) that were immunoreactive with antibody 6E10 (recognizing residues 1–17 of Aβ1–42 peptide) in the R/R samples but not WT samples. Each lane for WT and R/R cultures represents extracts from three independent cultures. hAPP = holo APP; βCTF = β C-terminal fragment. Asterisk denotes the presence of non-specific bands detected with the human-specific 6E10 antibody. **B**. Primary cortical neurons (21 DIV) from WT and R/R mice were prepared and grown for 24 h in the presence of fresh NB media with (+) or without (-) the B27 serum supplement. Immunoprecipitatation of conditioned media with antibody 6E10, followed by Western blot analysis with the anti-Aβ oligomer specific antibody, NU-2 revealed the presence of multiple NU-2 reactive bands between 30–40 kDa (arrows) that were present in the R/R (lanes 5 and 6), but not WT (lanes 2 and 3) samples. Lanes 1 and 4 shows total input lysates. **C-D**. Aβ oligomers are secreted by R/R neurons. Cultured primary cortical neurons (21 DIV) from wild-type (WT) and homozygous R1.40 (R/R) embryos were double labeled with MAP2 (green in **C **and **D**) or Aβ oligomer specific antibodies NU-1 (red in **C**) or NU-2 (red in **D**). Merged images are shown in right panel. NU-1 and NU-2 immunreactivities were specifically observed in R/R, but not WT, neurons. Scale bar 30 μm.

To determine whether Aβ oligomers are generated in the primary cortical neurons derived from the R/R mice, LDS soluble cell lysates from three independent cultures of non-transgenic WT and R/R neurons were analyzed via Western blot analysis with the human-specific anti-Aβ antibody 6E10. Western blot analysis revealed the production of human holo-APP in R/R lysates but not in lysates from WT cultures (Figure 5A). In addition, R/R lysates displayed the presence of multiple 6E10 reactive bands migrating between 14 kDa and 38 kDa that were absent in WT lysates (arrows, Figure 5A). These bands were also recognized by an anti-Aβ oligomer specific antibody, NU-1 [34], but were not recognized by antibodies specific to the N – (22C11) and C-terminus (CT15) of APP (data not shown).

To further examine whether the neurons from R/R embryos also secrete Aβ oligomers, immunoprecipitation analysis on conditioned media from WT and R/R neurons was performed using antibody 6E10 followed by Western blot with Aβ oligomer-specific monoclonal antibody NU-2 [34]. Similar to that observed for neuronal cell lysates, 6E10 immunoprecipitated, NU-2-immunoreactive 14–38 kDa bands were observed in conditioned media from R/R neurons, but not in WT controls (Figure 5B-R/R). The NU-2 immunoreactive bands were also discernable in the total input lysate from R/R but not WT cultures (Figure 5B-lanes 1 and 4).

To further confirm that R/R neurons were generating higher molecular weight aggregates containing Aβ, we performed double immunofluorescence on both WT and R/R neurons with MAP2 and oligomer specific antibodies NU-1 or NU-2. Notably, the R/R neurons, and not the WT neurons exhibited NU-1 and NU-2 immunoreactivity (Figure 5C and 5D). To examine the relative levels of Aβ generated from the R/R cultures in comparison to the levels of exogenous Aβ oligomers required for induction of neuronal CCEs, we quantified the levels of both cell associated and secreted Aβ (1–40) by ELISA. Notably, the levels of Aβ (1–40) were approximately three orders of magnitude lower in R/R conditioned media than the lowest concentration of synthetic Aβ required to significantly induce neuronal CCEs and process retraction when added exogenously (2.0 μg/ml versus 2.5 ng/ml; data not shown). Taken together, our data suggests that R/R neurons generate low levels of cell associated and secreted high molecular weight aggregates containing Aβ that may account for the elevated induction of neuronal CCEs and process retraction observed in these cells.

### Alterations in the PI3K-Akt-mTOR pathway following Aβ oligomer treatment

There is increasing evidence that the PI3K-Akt-mTOR pathway is altered upon Aβ stimulation and in human AD. First, this pathway acts downstream of many cell surface receptors to which Aβ has been demonstrated to interact with including the alpha 7 nicotinic acetylcholine receptor (CHRNA7), N-methyl-D-aspartic acid receptors [35,36] and insulin receptors [20]. Second, in AD cases, alterations in Akt phosphorylation correlate with the staging and severity of the disease [37]. These studies, together with the established role for mTOR as a downstream target for Akt in regulating growth factor mediated cell survival and proliferation (reviewed in [38]) suggested that the PI3K-Akt-mTOR pathway could be responsible for the induction of neuronal CCEs following Aβ oligomer exposure.

To examine if the exposure of synthetic preparations of Aβ oligomers to wild-type neurons elevates the levels of pAkt, we treated primary neurons with increasing concentrations of Aβ monomers and Aβ oligomers and the levels of pAkt and p-mTOR were determined by In-Cell Western (ICW) analysis. High-density primary neuronal cultures were prepared in 96 well plates, exposed to various concentrations of Aβ monomers and Aβ oligomers for 24 hours and examined by ICW for pAkt and p-mTOR staining. Tubulin was utilized as a control for normalization and values expressed as pAkt/tubulin and p-mTOR/tubulin ratios.

**Figure 6:**
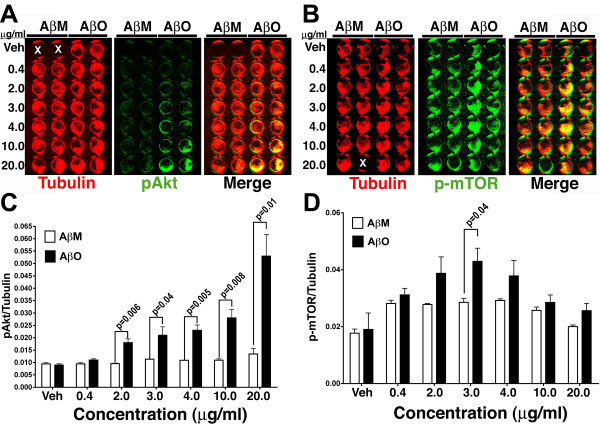
**Aβ oligomer treatment increases the levels of pAkt and p-mTOR in primary neurons**. **A**. Cultured primary cortical neurons (21 DIV) plated in 96-well plates were treated with either vehicle (Veh) or increasing concentrations (0.4, 2.0, 3.0, 4.0, 10 and 20 μg/ml) of either Aβ monomer (AβM) or Aβ oligomer (AβO) for 24 h. Following fixation, In-Cell Western analysis, using primary antibodies against tubulin and phosphorylated-Akt (pAkt^S473^) followed by infrared-conjugated secondary antibodies, revealed an apparent increase in pAkt levels in AβO treated cultures. **C**. Quantification of the pAkt/Tubulin ratio in these cultures revealed a dose-dependent statistically significant increase in AβO treated cultures at AβO concentrations above 2.0 μg/ml (2.0 μg/ml-p = 0.006; 3.0 μg/ml -p = 0.04; 4.0 μg/ml -p = 0.005; 10 μg/ml -p = 0.008; 20.0 μg/ml -p = 0.01; mean ± SD; n = 4; unpaired *t *test), while exposure to AβM did not reveal any alterations in the pAkt/Tubulin ratio. All comparisons were made against AβM at respective concentrations. **B**. In-Cell Western analysis with primary antibodies against tubulin and phosphorylated-mTOR (p-mTOR^S2448^) revealed an apparent increase in the levels of p-mTOR in the middle concentration ranges (2.0 – 4.0 μg/ml). **D**. Quantification of the p-mTOR/tubulin ratios revealed a statistically significant (3.0 μg/ml -p = 0.04; unpaired *t *test; mean ± SD; n = 4) increase in AβO exposed cultures when compared to AβM exposed cultures at similar concentrations. At elevated concentrations of AβO (> 4.0 μg/ml), there was no significant increase in the p-mTOR/tubulin ratio. 'X' denotes wells with no cells (in panel **A **and **B**).

The data demonstrates that the relative levels of pAkt were elevated upon exposure to increasing concentration of Aβ oligomers, but not Aβ monomers (Figure 6A and 6C). Neurons exposed to Aβ oligomers at a concentration of 2.0 μg/ml exhibited a statistically significant increase in pAkt levels that further increased to an approximately five-fold induction of pAkt upon exposure to 20.0 μg/ml of Aβ oligomers (Figure 4C). By contrast, exposure to Aβ monomers did not result in significantly altered levels of pAkt at any of the concentrations examined (Figure 6A and 6C). Notably, the concentration dependence of pAkt levels in response to Aβ oligomer treatment was similar to that observed for induction of neuronal CCEs (Figure 2N).

The levels of p-mTOR also increased upon exposure to Aβ oligomers. However, unlike pAkt levels, exposure of neurons to increasing concentrations of Aβ oligomers resulted in a bell-shaped curve, with statistically significant increase in p-mTOR at the 3.0 μg/ml concentrations (0.028 ± 0.0025 for AβM vs 0.0429 ± 0.008 for AβO) (Figure 6B and 6D) that dropped to basal levels upon exposure to higher concentrations of Aβ oligomers (4.0, 10.0 and 20 μg/ml). Meanwhile, exposure of neurons to Aβ monomers did not alter p-mTOR levels. The activation patterns of phospho-Akt and phospho-mTOR measured by ICW following Aβ treatments are consistent with earlier reports on the effect of Aβ_1–42 _on the activation of PI3K pathway [39,23]. Wei et al., (2002) have observed that treatment of SH-SY5Y cells with 4 μM (equivalent of 15 μg/ml) of aggregated Aβ_1–42 _activated Akt and this activation was sustained over 24 h period [39]. Similarly, Lafay-Chebassier et al., (2005) have observed aggregated Aβ_1–42 _at a high concentration (20 μM or equivalent of 80 μg/ml)) down-regulated the p-mTOR levels in differentiated Neuro-2a cells [23]. Together these results suggest that molecular components of PI3K pathway are sensitive to Aβ treatment.

**Figure 7:**
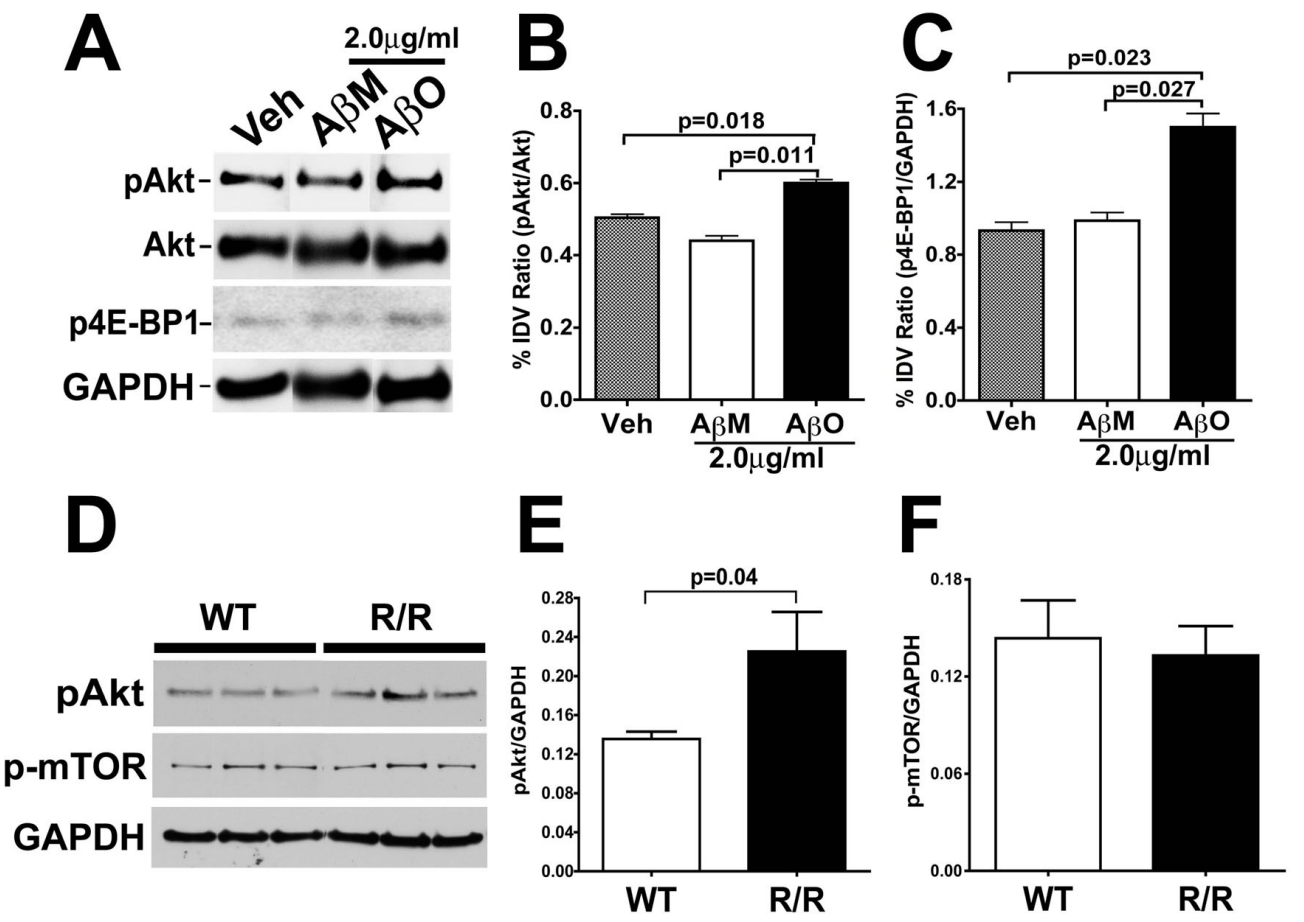
**Elevation of pAkt levels in primary neurons treated with Aβ oligomers and in neurons from the R1.40 transgenic mouse model of AD**. **A**. Cultured primary cortical neurons (21 DIV) were treated with vehicle (Veh), 2.0 μg/ml Aβ monomers (AβM) or 2.0 μg/ml Aβ oligomers (AβO) for 24 hours. Western blot analysis of cell extracts with antibodies against pAkt^S473^, total Akt, p4E-BP1^S65 ^and GAPDH revealed a slight increase in the levels of pAkt and p4E-BP1 in AβO treated cultures when compared to vehicle or AβM treated cultures. **B-C**. Quantification of the ratios of pAkt/total Akt (**B**) and p4E-BP1/GAPDH (**C**) revealed statistically significant increases upon exposure to AβO (2.0 μg/ml) when compared to AβM treatment (2.0 μg/ml) or vehicle controls (p < 0.05 for AβO versus vehicle or AβM; unpaired *t *test; mean ± SEM; n = 3 independent treatments). **D**. Western blot analysis of detergent soluble cell extracts of from cultured primary cortical neurons (21 DIV) from non-transgenic (WT) and homozygous R1.40 (R/R) mice with antibodies against pAkt (pAkt^S473^), p-mTOR (p-mTOR^S2448^) and GAPDH revealed elevated levels of pAkt (pAkt^S473^) but not p-mTOR in the R/R neurons when compared to WT neurons. **E-F **Quantification of the ratios of pAkt/GAPDH (**E**) and p-mTOR/GAPDH (**F**) revealed statistically significant increases in the levels of pAkt/GAPDH (p = 0.04; unpaired *t *test; mean ± SEM; n = 3 independent cultures) but not p-mTOR/GAPDH (p = 0.74; unpaired *t *test; mean ± SEM; n = 3 independent cultures) in cell lysates form the R/R cultures.

To confirm the ICW analysis of pAkt levels, conventional Western blot analysis was performed on cell lysates of neurons exposed to 2.0 μg/ml of Aβ oligomers, a concentration that was sufficient for the induction of neuronal CCEs. Western blots were probed with antibodies against pAkt (Ser 473) and total Akt and quantified for the relative levels of pAkt/Akt (Figure 7A and 7B). Our results demonstrate a relatively modest, but statistically significant increase in the pAkt/Akt ratio upon exposure to 2.0 μg/ml Aβ oligomers, with a 13% increase in the pAkt/Akt ratio following Aβ oligomers treatment compared to vehicle (0.6 ± 0.0096 vs 0.504 ± 0.009) and a 23% increase when compared to Aβ monomer treatment (0.6 ± 0.0096 vs 0.44 ± 0.014). These results confirm our ICW results and together demonstrate that exposure of neurons to Aβ oligomers results in altered pAkt and p-mTOR levels that correlate with the presence of neuronal CCEs as well as MAP2+ process loss.

To determine whether the downstream effectors of mTOR activation were also altered upon Aβ oligomer treatment, the levels of translation inhibitor 4E-BP1(inhibited by mTOR mediated phosphorylation) were examined following Aβ oligomer treatment at a concentration of 2.0 μg/ml. Our results demonstrate a statistically significant, 30–50% increase in the p4E-BP1^S65^/GAPDH ratio upon exposure to 2.0 μg/ml Aβ oligomers compared to vehicle or Aβ monomer exposure (Figure 7A and 7C), suggesting alterations in the downstream targets within mTOR pathway by Aβ oligomers.

### Alterations in the PI3K-Akt-mTOR pathway in R1.40 (R/R) neurons

To examine whether R/R neurons, which exhibit elevated levels of neuronal CCEs and also secrete Aβ oligomers, also display alterations in PI3K-Akt-mTOR pathway, the levels of pAkt and p-mTOR in WT and R1.40 neuronal lysates was determined. Western blot analysis with an antibody against pAkt revealed significantly elevated levels of pAkt in R/R samples when compared to WT samples (0.1355 ± 0.007 versus 0.225 ± 0.04 for WT and R/R respectively) (Figure 7D and 7E). On the other hand, Western blot analysis with antibodies against p-mTOR did not reveal any significant differences in the levels of p-mTOR/GAPDH between WT and R/R (Figure 7D and 7F). These results suggest that R/R neurons, which produce endogenous oligomeric Aβ species and exhibit elevated CCEs, also exhibit alterations in the Akt pathway.

### PI3K, Akt and mTOR inhibition prevents Aβ oligomer-induced neuronal CCEs

**Figure 8:**
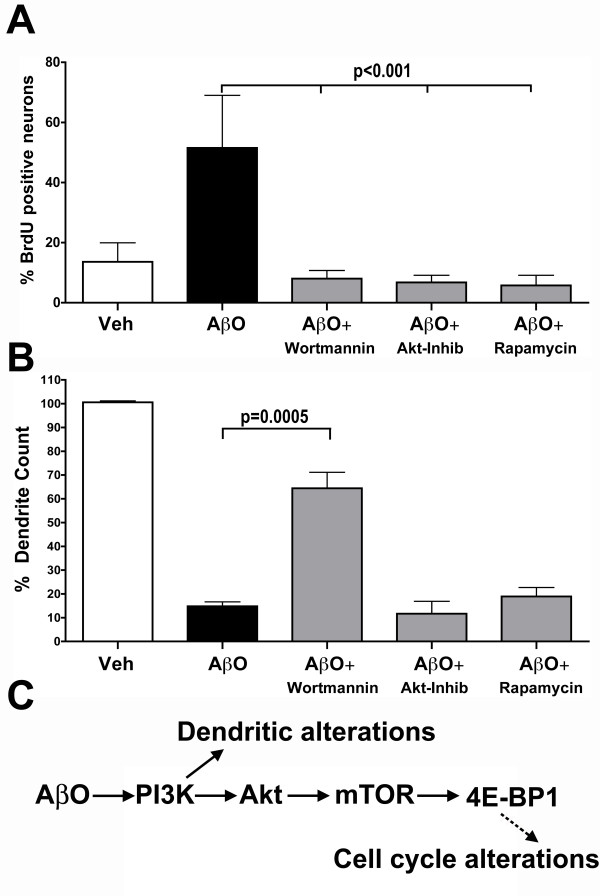
**Aβ oligomer induced neuronal CCEs are blocked with inhibitors of the PI3K/Akt/mTOR pathway**. **A**. Cultured primary cortical neurons (21 DIV) were pretreated for 30 min with the PI3K inhibitor wortmannin (100 nM), an Akt inhibitor (100 nM) and the mTOR inhibitor rapamycin (1 μM), followed by exposure to 2.0 μg/ml of Aβ oligomers (AβO) for 24 hours in the presence of BrdU. Following fixation, cells were stained with specific antibodies against BrdU and MAP2. Quantification of the BrdU positive/MAP2 positive cells demonstrated a statistically significant decrease in the percentage of neurons positive for BrdU with all three inhibitors (p < 0.001 for AβO vs AβO+wortmannin, AβO+Akt inhibitor and AβO+rapamycin; one-way ANOVA with Tukey multiple comparison test for pair-wise comparisons; mean ± SEM; n = 4 independent treatments per group). **B**. Quantification of the loss of MAP2 positive processes upon treatment with AβO in the presence of the PI3K, Akt and mTOR inhibitors was determined via automated image processing and revealed a statistically significant increase in the number of MAP2 positive processes upon pre-treatment with the PI3K inhibitor (p = 0.0005; unpaired *t *test; mean ± SEM; n = 4 independent treatments), but not the Akt or mTOR inhibitors when compared to AβO treatment alone. **C**. Diagram outlining a potential pathway underlining the effects of Aβ oligomers on neuronal process retraction and CCEs. Our data suggest that Aβ oligomers activate the PI3K-Akt-mTOR signaling pathway. Activation of PI3K causes phosphorylation of Akt at Ser473, which in turn activates mTOR via phosphorylation at Ser2448. Activation of mTOR results in induction of cell proliferation by down regulation of 4E-BP1 via inhibitory phosphorylation at ser65. Interestingly, our data suggest that the dendritic alterations induced by Aβ oligomers is blocked via inhibition of PI3K via as yet to be identified downstream effectors.

To provide evidence that the PI3K-Akt-mTOR pathway is directly involved in the Aβ oligomer-induced neuronal CCEs, WT primary cortical neurons were incubated with specific, cell-permeable, irreversible pharmacological inhibitors (Wortmannin for PI3K, Akt-inhibitor for Akt and Rapamycin for mTOR) 30 min prior to addition of Aβ oligomers (2.0 μg/ml) in the presence of BrdU. The number of BrdU+ MAP2+ cells was quantified for each treatment group in triplicates. Strikingly, pre-treatment of primary cortical neurons with PI3K, Akt or mTOR inhibitors resulted in over 80% reduction in the number of BrdU positive neurons compared to Aβ oligomer exposure alone (Figure 8A). This decrease in BrdU incorporation was not merely due to fewer numbers of cells as indicated by similar numbers of MAP2+ cells and DAPI stained nuclei and also the inhibitors alone did not have any direct effect on BrdU incorporation (data not shown). This suggests that the inhibition of several steps within the PI3K/Akt/mTOR pathway blocks Aβ oligomer induced neuronal CCEs.

### Inhibition of PI3K prevents Aβ oligomer-induced neuronal process loss

Exposure of primary neurons to Aβ oligomers results in neuronal CCEs that are correlated with loss of neuronal processes (Figure 3). To examine whether the PI3K/Akt/mTOR pathway is involved in the Aβ oligomer-induced neuronal process loss, the total number of MAP2+ processes was quantified in presence or absence of the wortmannin, Akt inhibitor and rapamycin. Unlike the results obtained for induction of neuronal CCEs, only the PI3K inhibitor significantly rescued the Aβ oligomer-induced neuronal process loss. A 76% percent rescue in the total dendrite count (14.79 ± 1.89 for AβO versus 64.5 ± 6.67 for AβO+wortmannin) was observed with wortmannin. While the Akt inhibitor and rapamycin were indistinguishable from Aβ oligomer treated cultures (Figure 8B). Together, these results suggest that neuronal CCEs and neuronal process loss are part of a coordinated cellular response to Aβ oligomers that involves the common upstream PI3K pathway, while neuronal CCEs, but not neuronal process loss acts via the downstream Akt and mTOR pathway.

## Discussion

The current studies offer significant insights into the mechanisms underlying the induction of neuronal cell cycle abnormalities observed in human AD. Neuronal CCEs are an early and robust marker for neurons subject to degeneration in AD. Although aberrant neuronal cell cycle is closely linked to AD pathogenesis [3-6], the cellular/molecular mechanisms responsible for the induction of neuronal CCEs remains unclear. Recent findings from our laboratory has demonstrated the induction of neuronal CCEs in the genomic-based R1.40 mouse model of AD that occurs substantially prior to Aβ deposition, correlate with the relative levels of Aβ generated, dependent upon the amyloidogenic processing of APP and can be induced in primary neurons with crude preparations of Aβ oligomers [9]. The studies described here provide a further confirmation of the role of Aβ oligomers in the induction of neuronal CCEs as well as neuronal process retraction and implicates the role of the PI3K/Akt/mTOR signaling pathway in the specific induction of neuronal CCEs.

The current findings utilized relatively pure preparations of Aβ monomers and Aβ oligomers. The crude preparations of Aβ that we used in our earlier studies was based on an established protocol [32], with which we and others have observed different biological effects on neuronal CCEs [9], synapse and dendrite integrity [15], inhibition of LTP [12] and neuronal viability [32]. However, the Aβ monomers prepared via these protocols inevitably contains low-n Aβ oligomers (trimers and tetramers), which confounds the interpretation regarding the specific effects of Aβ oligomers on neurons. To overcome this, the current studies prepared mixtures of synthetic Aβ monomer and oligomers via aging of the samples for 24 hours and subsequently fractioning the sample into Aβ monomers and oligomers using size exclusion chromatography. Indeed, SEC fractionation yielded two distinct peaks for Aβ oligomers and Aβ monomers which when separated by SDS-PAGE yielded a single distinct band at 4.5 kDa for purified Aβ monomers and multiple oligomeric as well as a monomeric Aβ bands for the purified Aβ oligomers. These results are entirely consistent with previous studies examining the preparation and purification of synthetic Aβ species via SEC and SDS-PAGE, in which SDS-PAGE appears to alter the migration pattern of the Aβ species [26-28].

Another significant issue regarding the postulated biological effects of Aβ monomers and oligomers concerns the potential for induction of altered Aβ species during the experimental paradigm. In the current experiments, a detailed analysis of the Aβ monomers and oligomers from the media of the cells via SDS-PAGE revealed no significant alterations in the migration pattern of the Aβ monomers and oligomers during the 24 hour incubation utilized in the current studies.

These studies confirmed that SEC purified Aβ oligomers, and not similar concentrations of SEC purified Aβ monomers or preparations of Aβ fibrils were capable of inducing neuronal CCEs in a concentration-dependent manner. Quantification of BrdU incorporation in these cultures, revealed that neurons treated with 4.0 μg/ml of Aβ oligomers exhibited ~40% of the neurons incorporating BrdU compared to ~5–10% for the Aβ monomers, Aβ fibrils or vehicle control. In addition to increased BrdU incorporation these studies also confirmed increased expression of the S-phase cell cycle protein PCNA.

While several previous studies have suggested that fibrillar Aβ can directly induce CCEs [29,30,40], these experiments were performed in differentiated SHSY5Y cells [29,40], the concentration of Aβ utilized to induce CCEs was in the μM range [30] (versus effects observed with as little as 2.0 μg/ml or equivalent of 500 nM in the present study) with longer incubation times (previous studies utilized 72 hour exposure versus 24 hour exposure in the present study) [30]. Moreover, in the earlier reports the Aβ preparations were likely crude mixtures of monomers, oligomers and fibrils (versus the SEC purified preparations in the present study). Numerous other studies have also documented biological effects of Aβ oligomers in the nM (< 4.0 μg/ml) range (similar to those used in the present study) including synaptic loss [15], inhibition of LTP [12] and impairment of memory in animal models of AD [19]. These studies suggest that Aβ oligomerization can induce biological effects in certain neuronal populations, including expression of certain cell cycle proteins, DNA replication as well as alterations in dendritic/synaptic structures and general morphology.

Our results demonstrate that purified Aβ oligomers induce both dendritic loss and CCEs within the same groups of neurons. Intriguingly, several recent studies have demonstrated that Aβ oligomers can directly cause loss of synaptic proteins such as drebrin, PSD95, alterations in several neurotransmitter receptors including, nAChRs [41]; muscarinic receptors [42] and glutamate receptor trafficking [43], altered spine composition, morphology and density [15], as well as dendritic pathology [44] both *in vivo *and *in vitro*. Taken together with the results presented here, this supports the hypothesis that Aβ oligomers induce a common biological response that involves both dendrite loss and induction of neuronal CCEs.

We also demonstrated that the induction of CCEs is cell autonomous to primary cortical neurons from the R1.40 (R/R) mouse model, and is accompanied by the shortening of dendritic processes, In addition, we detected the presence of 17 to 38 kDa Aβ-containing oligomers in the R/R neurons, but not in control cultures. Notably, the levels of Aβ in the R/R neuronal cultures (2.5 ng/ml) is substantially less than that utilized to induce neuronal CCEs in wild-type neuronal cultures (2 μg/ml). However, as APP is expressed under the control of the human APP promoter in the R/R neurons and expressed from early embryonic stages [45,46], the R/R neurons will be chronically exposed to low-levels of Aβ species, as opposed to the acute 24 hour exposure of the 21 DIV wild-type neurons. In support of this idea, R/R neurons exhibit shorter neuronal processes even at earlier DIV (data not shown).

It should be noted that the induction of neuronal CCEs in the R/R neuronal cultures could be due to the elevated expression of human APP and its processed products, some of which have been demonstrated to participate in cellular adhesion and/or neuronal differentiation (reviewed in [47]). Future experiments will be required in order to determine whether the production of Aβ species in R/R neurons is directly responsible for the induction of CCEs and dendrite shortening, including the use of β- and γ-secretase inhibitors as well as R/R neurons lacking β-secretase.

While the precise molecular mechanisms underlying Aβ oligomer-driven induction of neuronal CCEs and dendrite loss is not completely clear, our data support a potential role of the PI3K/Akt/mTOR pathway. First, exposure of primary neurons to increasing concentrations of Aβ oligomers, but not Aβ monomers, resulted in increased levels of p-Akt^S473^. This is consistent with the earlier reports on ADDL induced phosphorylation of Akt at ser473 is associated with removal of dendritic insulin receptors and synaptotoxicity [20]. Second, exposure of primary neurons to Aβ oligomers also elevated levels of phospho-mTOR^s2448 ^and phospho-4E-BP1^s65^, downstream targets of Akt. While other reports have suggested either unaltered [48] or decreased [23] phospho-mTOR levels in APP^SL^/PSI KI mouse models of AD and following Aβ treatment in N2a cell lines respectively, an increase in mTOR phosphorylation has been reported in the AD brain [22,25]. The discrepancy between increased phospho-mTOR levels in our study and reduced phospho-mTOR levels in Aβ treated N2a cells [23] may be due to different preparations of Aβ peptide used (oligomeric Aβ at < 4.0 μg/ml or in 1000 nM concentration-our study; versus fibrillar Aβ at 20 μM or equivalent of 80 μg/ml -[23]). Finally, direct evidence for the participation of the PI3K/Akt/mTOR pathway in the induction of neuronal CCEs upon Aβ oligomer exposure was provided by demonstration that inhibition of either any one component of this pathway significantly inhibited BrdU incorporation. Interestingly, earlier reports, using pharmacological and genetic means, have demonstrated that PI3K/Akt does play a crucial role in the proliferation of neural progenitor cells by transducing intracellular signals from multiple mitogens including fibroblast growth factor-2, sonic hedgehog, and insulin-like growth factor [49,50]. In addition, inhibition of mTOR in cancerous tissue has been shown to decrease cell proliferation (reviewed in [51]).

In summary, our results demonstrate that purified Aβ oligomers, but not Aβ monomers or fibrils, induce neuronal CCEs by altering components of the PI3K/Akt/mTOR pathway (Figure 8C). Further studies are required to examine the detailed molecular mechanisms underlying the various biological effects of Aβ oligomers and relate this to specific biochemical and pathological alterations that occur in AD. Significantly, there are several components of the PI3K/Akt/mTOR pathway that can be approached therapeutically to test the significance of neuronal CCEs in mouse models as well as in human AD.

## Materials and methods

### Antibodies

#### For immunocytochemistry

a mouse monoclonal antibody against microtubule-associated protein 2 (1:1000; MAP2; Sigma, St. Louis, MO) and a rat monoclonal antibody against BrdU (1:1000; Abcam, Cambridge, MA), mouse monoclonal antibodies against Aβ oligomers/ADDLs (NU-1 or NU-2; 1:100) were utilized [34].

#### For western immunoblots

a mouse monoclonal antibody against Aβ oligomers/ADDLs (NU-2; 1:2000) [34], a mouse monoclonal antibody against human Aβ (6E10; 1:5000; #SIG-39320; Covance, USA) a rabbit polyclonal antibody against phospho-Akt^Ser473 ^(1:1000; #9271, Cell Signaling, Danvers, MA), a rabbit polyclonal against total Akt (1:1000; #9272, Cell Signaling, Danvers, MA) a rabbit monoclonal antibody against phospho-mTOR^Ser2448 ^(1:200; clone 49F9, #2976, Cell Signaling, Danvers, MA), a rabbit polyclonal antibody against phospho-4E-BP1^S65 ^(1:1000; #9451; Cell Signaling, Danvers, MA), a mouse monoclonal antibody against PCNA (1:1000; #SC-56, Santa Cruz Biotechnologies, Santa Cruz, CA), a mouse monoclonal antibody against GAPDH (1:20,000; #MAB374; Millipore Corporation, USA) and a rabbit polyclonal antibody against tubulin (1:200; #T3526; Sigma, St. Louis, MO) were utilized.

#### For in-cell westerns

A rabbit polyclonal antibody against phospho-Akt^Ser473 ^(1:100), a rabbit monoclonal antibody against phospho-mTOR^Ser2448 ^(1:200), a mouse monoclonal antibody against tubulin (1:200; DM1A, NeoMarkers, Fremont, CA) or rabbit polyclonal antibody against tubulin (1:200; T3526; Sigma, St. Louis, MO) were utilized.

### *In vitro *preparation of Aβ_1–42 _monomeric and oligomeric samples

The method for preparations and purification of monomeric and oligomeric Aβ_1–42 _was based on Walsh et al., (1997) with minor modifications [52]. Briefly, synthetic Aβ_1–42 _peptide was prepared using standard fluorenylmethyloxycarbonyl chloride chemistry on an automated 433A synthesizer (Applied Biosystems), and was purified by HPLC using a Hamilton PRP-3 preparative column. The purified peptide was characterized by mass spectrometry and nuclear magnetic resonance spectroscopy [53]. A stock solution of monomeric peptide was prepared in 10 mM sodium hydroxide solution (pH 10.5), from which aliquots were removed and diluted into cold (0–5°C) sodium phosphate buffer at pH 7.4. The pH was maintained at 7.4 using cold dilute sodium hydroxide or trifluoroacetic acid solutions. The sample was allowed to age at room temperature, during which aggregation occurred, and the extent of aggregation was monitored by size exclusion chromatography (SEC) using a Sephadex™ G-75 size exclusion column at a flow rate of 0.5 ml/min. The column was equilibrated with 10 mM sodium phosphate buffer pH 7.4, which was filtered and degassed to eliminate any particulates or gas that may be present. Monomeric and oligomeric fractions were collected and kept cold (0–5°C) until needed for testing on cells. The oligomer sample eluted first (12–15 min), whereas the monomer eluted as a second band (25–30 min). In this modified method, Aβ fibrils are excluded from the Sephadex G-75 column since they have molecular mass in excess of 100 kDa, which is the exclusion limit of the column. Therefore, Aβ fibrils are not present in our SEC monomeric or SEC oligomeric samples as previously reported [52]. In addition, we confirmed the absence of Aβ_1–42 _fibrils in our SEC samples by SDS-PAGE analysis (Figure 1B).

The Aβ_1–42 _concentration of the eluted fractions was determined using Beers law (A = εLc,) where A is the absorbance measured for the sample at 220 nm, and e is the extinction coefficient expressed as 50,000 M^-1^cm^-1 ^at 220 nm [54]. The extinction coefficient was determined at 214-, 220-, and 280-nm [55].

The preparation of synthetic Aβ_1–42 _fibrils was prepared based on the published protocol with minor modification [32]. Briefly, HFIP-treated lyophilized Aβ_1–42 _peptide (A-1163-1; rPeptide, Bogart, GA) was carefully and completely resuspended to 5 mM in 1% ammonium hydroxide (Sigma, St. Louis, MO) by pipette mixing followed by brief sonication. 5 mM samples were aliquoted and stored at -20°C until further use. The recombinant Aβ_1–42 _peptide was diluted to 100 μM (40 μg/ml) in 100 mM HCl and incubated at 37°C for 24 h to obtain Aβ_1–42 _fibrils. Fibrils were diluted in Neurobasal media prior to cell treatment.

### Primary cortical neuronal cultures and Aβ treatments

Embryonic cortical neurons (E16.5 C57BL/6 mouse embryos) from wild type-(WT) or homozygous R1.40 transgenics (R/R) were isolated by standard procedures [56]. Briefly, embryos were collected in ice-cold PBS glucose, and the cortical lobes dissected. The meninges were subsequently removed, and the cortices placed in 1× trypsin-EDTA and DNAse (1.6 units/embryo) for 15 min at 37°C. The tissue was removed from the trypsin solution and placed in DMEM with 10% fetal bovine serum to inactivate the trypsin, followed by transfer to Neurobasal media supplemented with B-27, penicillin-streptomycin (1×), and L-glutamine (0.3 mM) (NB-media). Tissue was triturated (10 times) through a 5 ml pipette and allowed to settle to the bottom of a 15 ml conical tube. Cells in the solution were removed and plated onto poly-L-lysine-coated glass coverslips (0.05 mg/ml) at a density of 40,000 cells/well in 24-well plates or 160,000 cells in 6-well plate. All cultures were grown for 21 days *in vitro *(DIV) before any treatment.

To assess the effect of monomeric and oligomeric Aβ_1–42 _on neuronal CCEs, the SEC fractions or the phosphate buffer (PB) as vehicle (control) were diluted serially in NB-media containing 10 μM BrdU and exposed to the neurons for 24 h. For the analysis of specific inhibitors, 21 DIV cortical neurons were pre-incubated for 30 min with following inhibitors; wortmannin (PI3K inhibitor, W1628, Sigma; final concentration of 100 nM)[57], Akt inhibitor (A6730, Sigma; final concentration of 1 μM)[58] or rapamycin (mTOR inhibitor, final concentration of 100 nM)[59] in presence or absence of 10 μM BrdU. The neurons were subsequently treated with Aβ oligomers (500 nM) for 24 h. To study the basal levels of neuronal CCEs in neurons from R1.40 mouse embryos, on 20 DIV, NB media was replaced with fresh NB-media containing 10 μM BrdU and incubated for 24 h. Cells were fixed with 4% paraformaldehyde in PB at room temperature for 30 min, washed and stored in phosphate buffered saline (PBS). All experiments were performed in triplicates and on cultures from three different litters.

### Immunocytochemistry

For visualization of BrdU incorporation, cells were treated with 2 N HCl for 30 min at 37°C, neutralized in 0.1 M sodium borate (pH 8.6) for 10 min and washed extensively in PBS (5 times). Cells were blocked with 5% normal goat serum in PBS with 0.4% Triton X-100 for 1 h at room temperature, incubated overnight at 4°C with the primary antibodies (in blocking buffer) against MAP2 and BrdU. Following 3 washes with PBS at room temperature (RT), cells were incubated with anti-mouse and anti-rat secondary antibodies conjugated to Alexa400 and Alexa546 (1:1000; Invitrogen, Eugene, OR) for 1 h at RT. Cells were subsequently washed with PBS and coverslipped with hard-set mounting media with DAPI (Vector Laboratories, Burlingame, CA).

### Quantification of cells positive for MAP2 and BrdU

The number of cells positive for BrdU only, MAP2 only or positive for both were quantified by scoring five random fields per treatment per each concentration on digital images obtained on a fluorescent microscope. By assigning total number of MAP2 positive cells per treatment as 100%, the percentage of total number of MAP2 positive and BrdU positive cells were calculated, normalized and expressed as mean ± SEM (n = 3). To quantify the percentage of MAP2 positive neurites per cell per treatment, the digital images was processed using the Image Pro Plus software package (Media Cybernetics, Bethesda, MD). First, the images were skeletonized with in-built morphological filter – "Thinning". This algorithm converts all MAP2 positive processes to 1-pixel thick open branches for accurate quantification. Thinned MAP2 positive photomicrographs were further processed with a specific Image Pro Plus macro ("Dendrites") that specifically quantifies the total number and the area of MAP2 positive processes per cell in each field. A dendrite, as defined by the Image Pro software, is a one pixel wide process off of a larger (two or more pixels wide) object (surface of the cell body). The "Dendrite" macro creates a mask on all the dendrites originating from different objects (cell body) in a visual field and quantifies total area occupied by the masked one pixel wide dendrites. The mean ± SEM (n = 3) of MAP2 positive neurites from five different fields per treatment were quantified and the percentage difference was determined by comparing with vehicle treated group that was assigned 100%. Data were statistically analyzed by an unpaired *t *test (GraphPad Prism, GraphPad Software Inc. San Diego, CA).

### Quantification of the total number and length of MAP2 positive processes per neuron

The total number of dendrites per MAP2 positive neuron and the mean dendritic length of 5 μm or more per BrdU positive and BrdU negative neurons were analyzed as above except that after "thinning" the images, the dendritic segments originating from one neuron and the total number of dendrites measuring 5 μm or more in length were manually quantified. At least, 5 visual fields per treatment were quantified and expressed as mean ± SEM. Each treatment was done in triplicates in three independent experiments. Data were statistically analyzed by an unpaired *t *test (GraphPad Prism).

### Immunoprecipitation

Immunoprecipitations from neuronal conditioned media was performed using a modified protocol of Sarkar et al., 2008 [60]. Briefly, 21 DIV conditioned media (CM) grown with or without B27 serum supplement for 24 h was collected, centrifuged at 1000 × g for 10 min to remove dead cells and then mixed with protease inhibitor cocktail (1:1000; Sigma Aldrich, St. Louis, MO). CM was concentrated via an Ultracel YM-10 (10,000 MWCO, Millipore, Bedford, MA) centricon filter column centrifuged at 3000 × g for 15 min at 4°C. CM was subsequently precleared by incubating with pre-blocked 10% protein-A-sepharose beads (GE Healthcare) for 1 h at 4°C. Aβ was immunoprecipitated by incubating pre-cleared CM with human specific monoclonal antibody 6E10 overnight at 4°C, then they were incubated with pre-blocked 10% Protein A sepharose for 1 h at 4°C, centrifuged at 1000 × g for 5 min. Following several washes, the immunoprecipitated proteins were resolved by sodium dodecyl sulfate-polyacrylamide gel electrophoresis (SDS-PAGE). Oligomeric species of Aβ in the immunoprecipitations were detected using an Aβ oligomer specific mouse monoclonal antibody NU-2 [34].

### Gel electrophoresis and Western blot analysis

SDS-PAGE and Western blot analysis were performed according to the manufacturer's protocols (Invitrogen, Carlsbad, CA). Samples were prepared in 1× lithium dodecyl sulfate (LDS) buffer, applied to 4–12% bis-Tris NuPAGE gels, resolved using MES running buffer and transferred to 0.45 μm polyvinylidene difluoride (PVDF) membranes. Membranes were subsequently blocked in 5% milk in PBS. Primary antibodies were diluted in 5% milk with 0.1% Tween 20. Following overnight incubation at 4°C with primary antibodies, blots were washed with PBS and detected utilizing anti-mouse or anti-rabbit secondary antibodies conjugated to horse-radish peroxidase (HRP) (1:20,000; #115-035-003 or #111-035-003, Jackson ImmunoResearch Laboratories, West Grove, PA) diluted in 5% milk with 0.1% Tween 20. To confirm equal protein loading, mouse anti-GAPDH or rabbit anti-tubulin antibodies were used. Bands were quantified utilizing the AlphaEaseFC™ Software (Ver 4.0.1 – a part of Alpha Innotech gel documentation system (Alpha Innotech Corporation, USA)).

### In-Cell western analysis

Expression of phospho-Akt/phospho-mTOR in primary cortical neurons was quantified using the infrared-based In-Cell Western (ICW) assay system as described by the manufacturer (Li-Cor Biosciences, Lincoln, NE). Briefly, primary cortical neurons were seeded into 96-well plates at a density of 5 × 10^4 ^cells/well. At 21 DIV, cells were treated with different concentrations of Aβ monomers or Aβ oligomers. After 24 h, cells were washed with PBS, fixed with 4% paraformaldehyde in PBS, blocked with 5% normal goat serum (in PBS with 0.4% Triton-X100) and incubated overnight at 4°C with different combinations of primary antibodies; a rabbit polyclonal antibody against phospho-Akt^Ser473 ^and a rabbit monoclonal antibody against phospho-mTOR^Ser2448^. For normalization, mouse anti-tubulin or rabbit anti-tubulin antibodies were used. Cells were subsequently incubated with secondary antibody mixtures (goat anti-mouse IRDye 700 and goat anti-rabbit IRDye 800 (1:200; Rockland Immunochemicals, Gilbertsville, PA) for 1 h at RT. Infrared signals were measured and quantified in an Odyssey infrared imaging system (Li-Cor Biosciences). The intensity of both channels was set at 8 and the focus offset was set at 3 mm for plate scanning. The integrated intensity ratio for pAkt/Tubulin or p-mTOR/Tubulin was calculated and the mean ± SEM for three independent experiments were used for statistical analysis.

## Competing interests

The authors declare that they have no competing interests.

## Authors' contributions

KB carried out the cell treatment experiments, analysis, interpretation and drafted the manuscript. MM and MZ provided SEC purified Aβ peptides. AC conducted process retraction experiment. KH and BTL participated in study design and coordination and helped to draft the manuscript. All authors read and approved the final manuscript.
